# Supplementary material for: Yeast Rpd3L histone deacetylase couples nutrient shifts to genome-wide chromatin reprogramming
Source: Cell Rep. Author manuscript; Available in PMC 2026 Sep 23. (PMC13599394; doi:10.1016/j.celrep.2026.117651)
Supplement: Supplementary file 1 [file NIHMS2199961-supplement-1.pdf]

Cell Reports, Volume *45*

Supplemental information

Yeast Rpd3L histone deacetylase couples nutrient shifts to genome-wide chromatin reprogramming

Saikat Bhattacharya, Benjamin M. Sutter, and Benjamin P. Tu

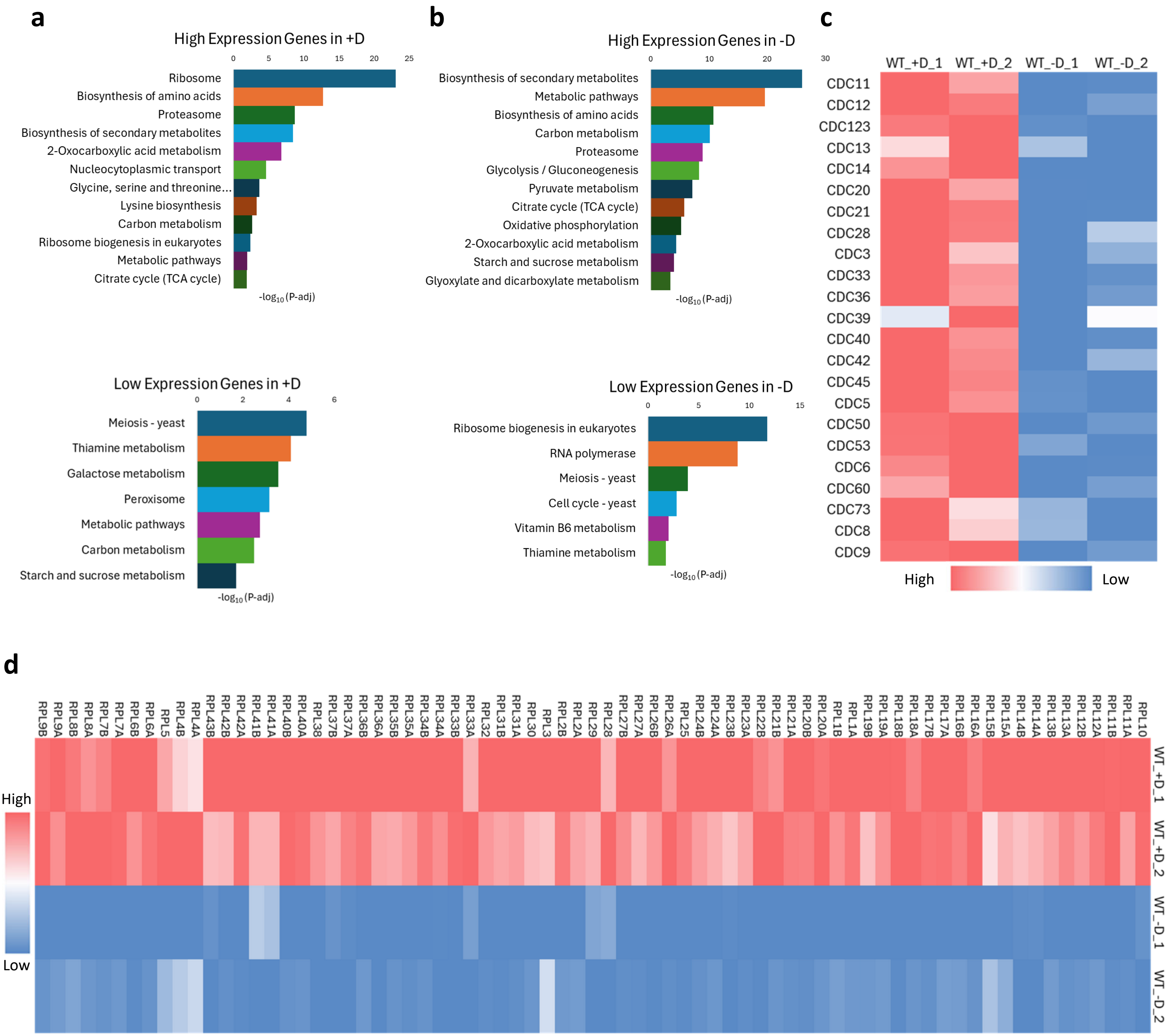

Supplementary figure S1: (a, b) KEGG analysis of highly or lowly expressed genes in glucose-replete (+D) or glucose-deplete (-D) media. (c, d) Heatmap showing the normalized counts of cell cycle (c) and ribosomal (d) genes from RNA-seq in +D and –D for the WT strain.

a

Class	HDAC	Complex/Localization
Class I (Rpd3-like)	Rpd3	Rpd3L, Rpd3S complexes
	Hos1	Nuclear
	Hos2	Set3C complex
	Hos3	Cytoplasm, nuclear periphery
Class II (Hda1-like)	Hda1	Hda1 complex (with Hda2/Hda3)
Class III (Sirtuin)	Sir2	SIR complex (Sir2/3/4)
	Hst1	Sum1/Rfm1/Hst1 complex
	Hst2	Cytoplasmic & nuclear
	Hst3	Nuclear
	Hst4	Nuclear

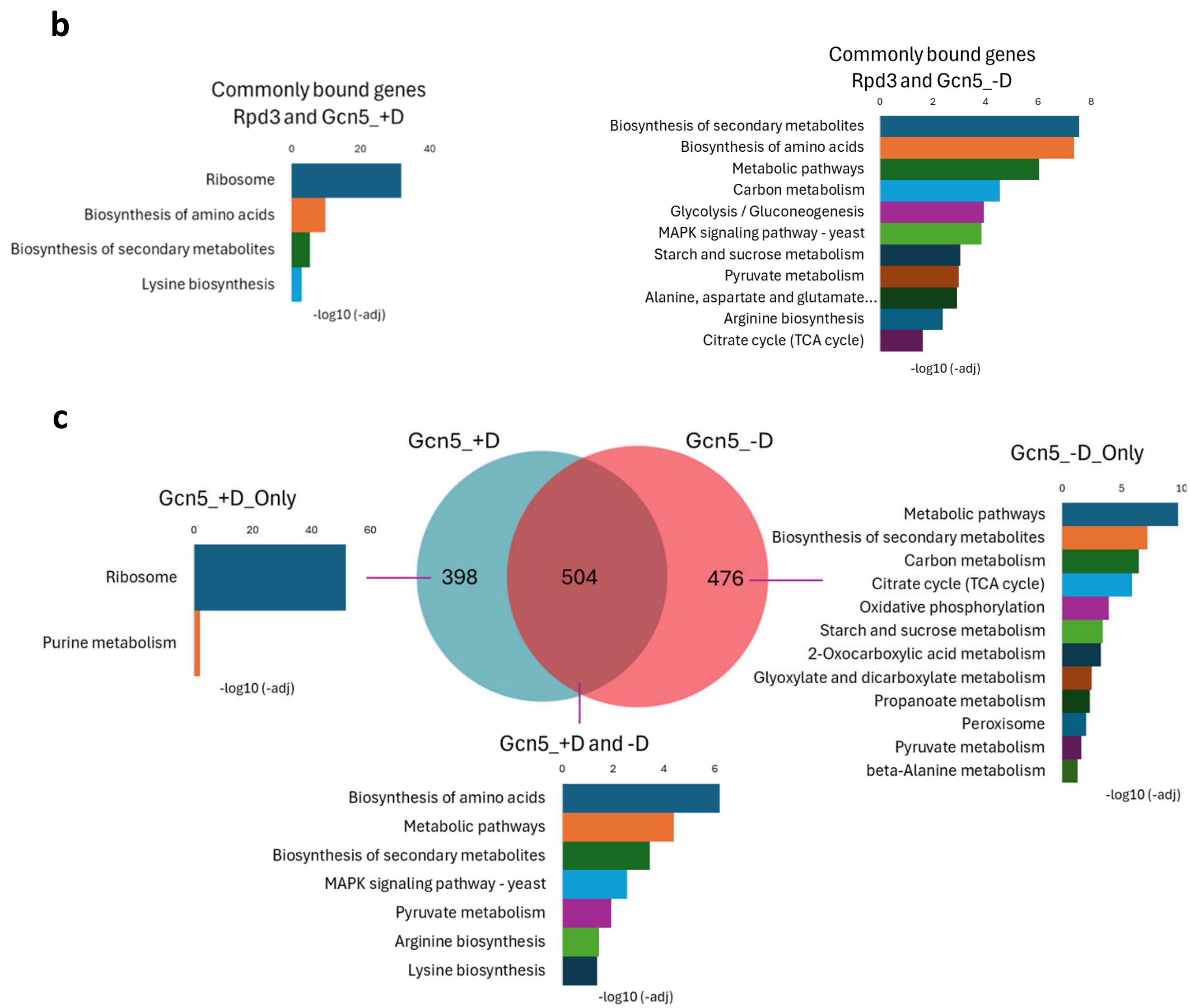

Supplementary figure S2: (a) HDACs in yeast. The HDACs highlighted in green were deleted from CEN.PK and the effect on H3K9ac level were tested by Western blotting. (b) KEGG analysis of commonly bound genes by Rpd3 and Gcn5 in +D and –D. (c) Venn diagram showing the overlap of genes that Gcn5 binds in +D vs -D. The three subsets were subjected to KEGG analysis, and the enriched pathways are shown.

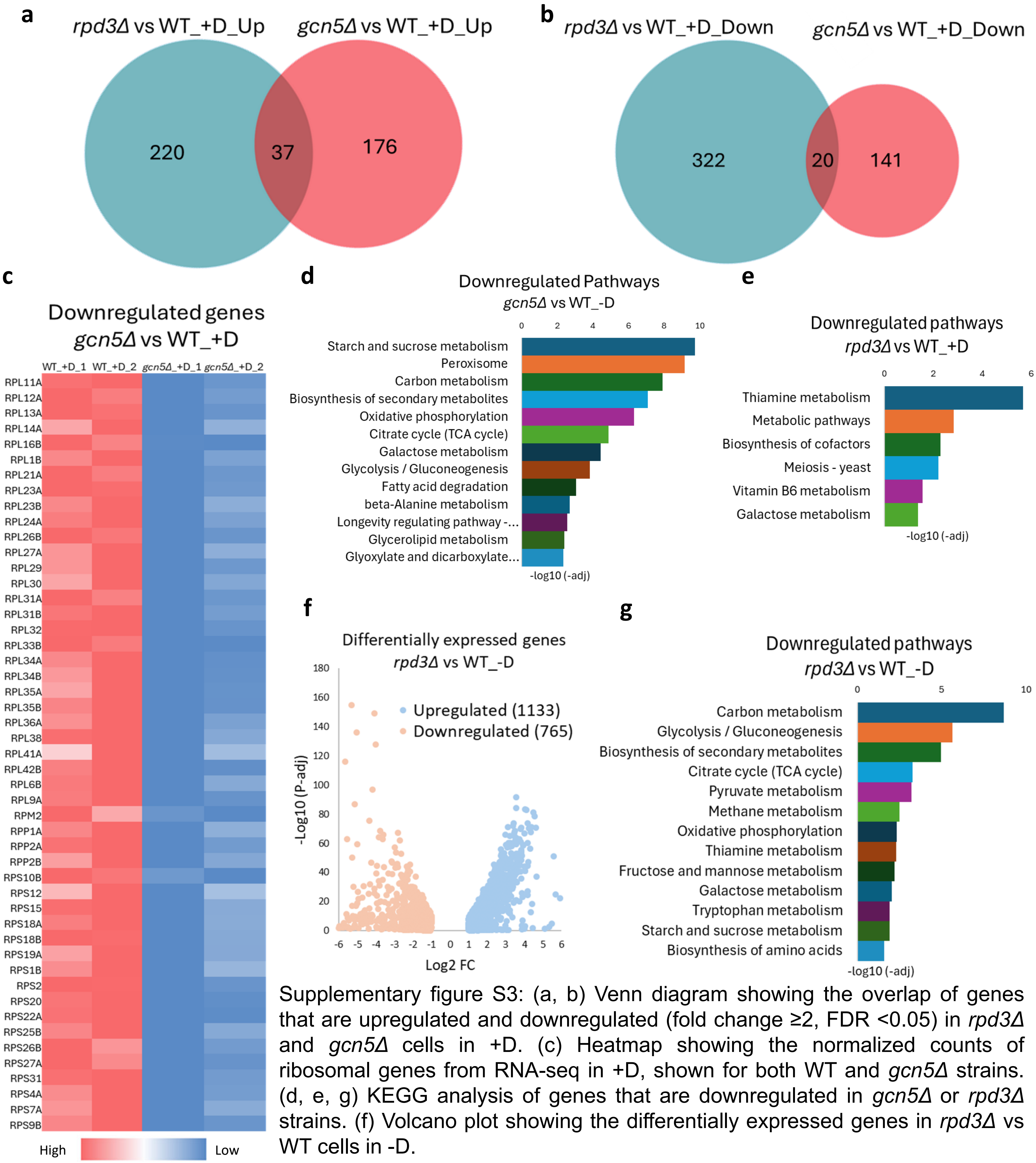

Supplementary figure S3: (a, b) Venn diagram showing the overlap of genes that are upregulated and downregulated (fold change ≥2, FDR <0.05) in *rpd3Δ* and *gcn5Δ* cells in +D. (c) Heatmap showing the normalized counts of ribosomal genes from RNA-seq in +D, shown for both WT and *gcn5Δ* strains. (d, e, g) KEGG analysis of genes that are downregulated in *gcn5Δ* or *rpd3Δ* strains. (f) Volcano plot showing the differentially expressed genes in *rpd3Δ* vs WT cells in -D.

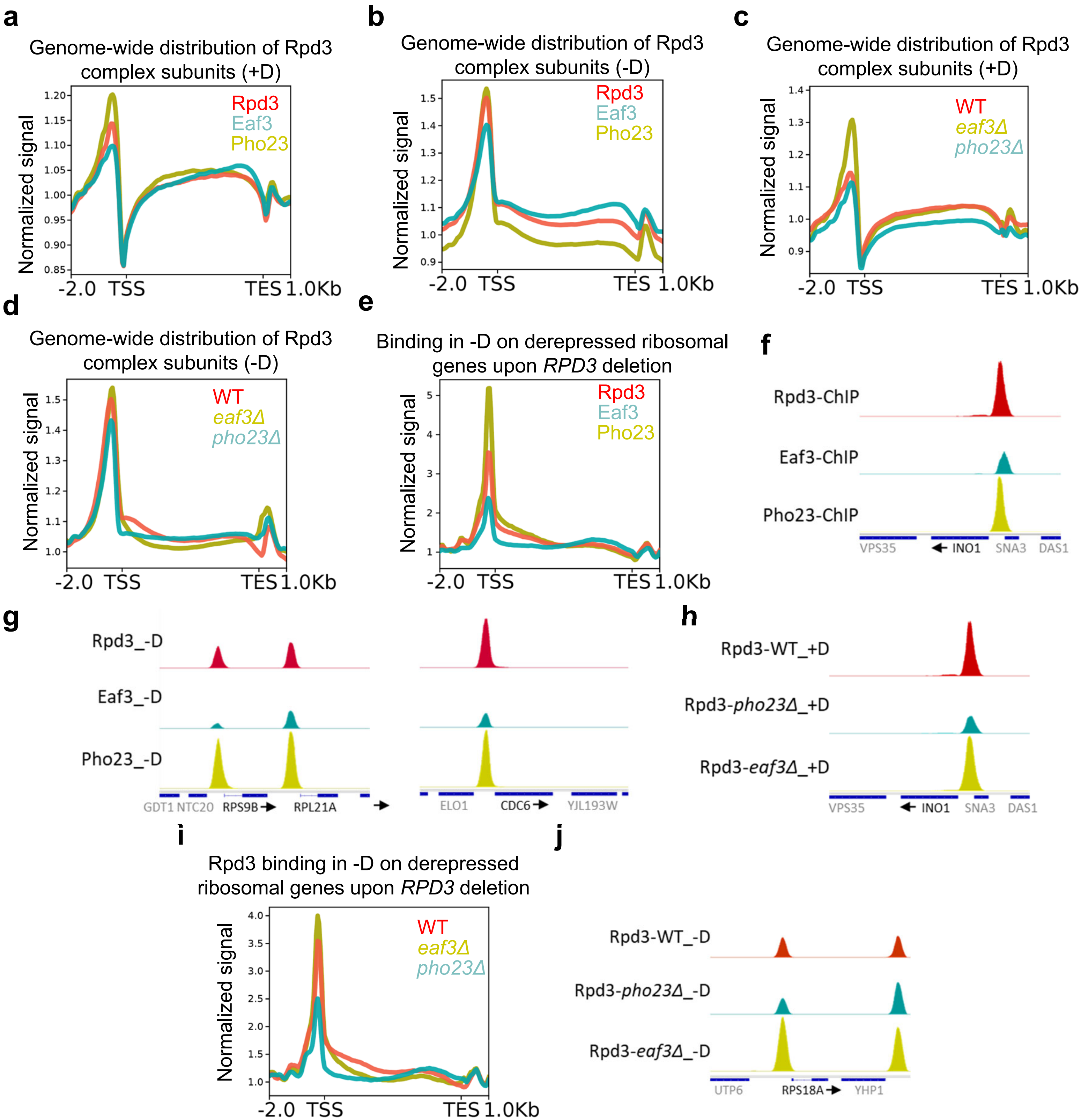

Supplementary figure S4: (a-e, i) Metagene profiles showing the genome-wide distribution of Rpd3, Eaf3, and Pho23 across all genes, as well as specifically at genes that are derepressed upon *RPD3* deletion in −D. (f-h, j) Representative genome browser tracks displaying ChIP-seq peaks for Rpd3, Eaf3, and Pho23 at selected loci.

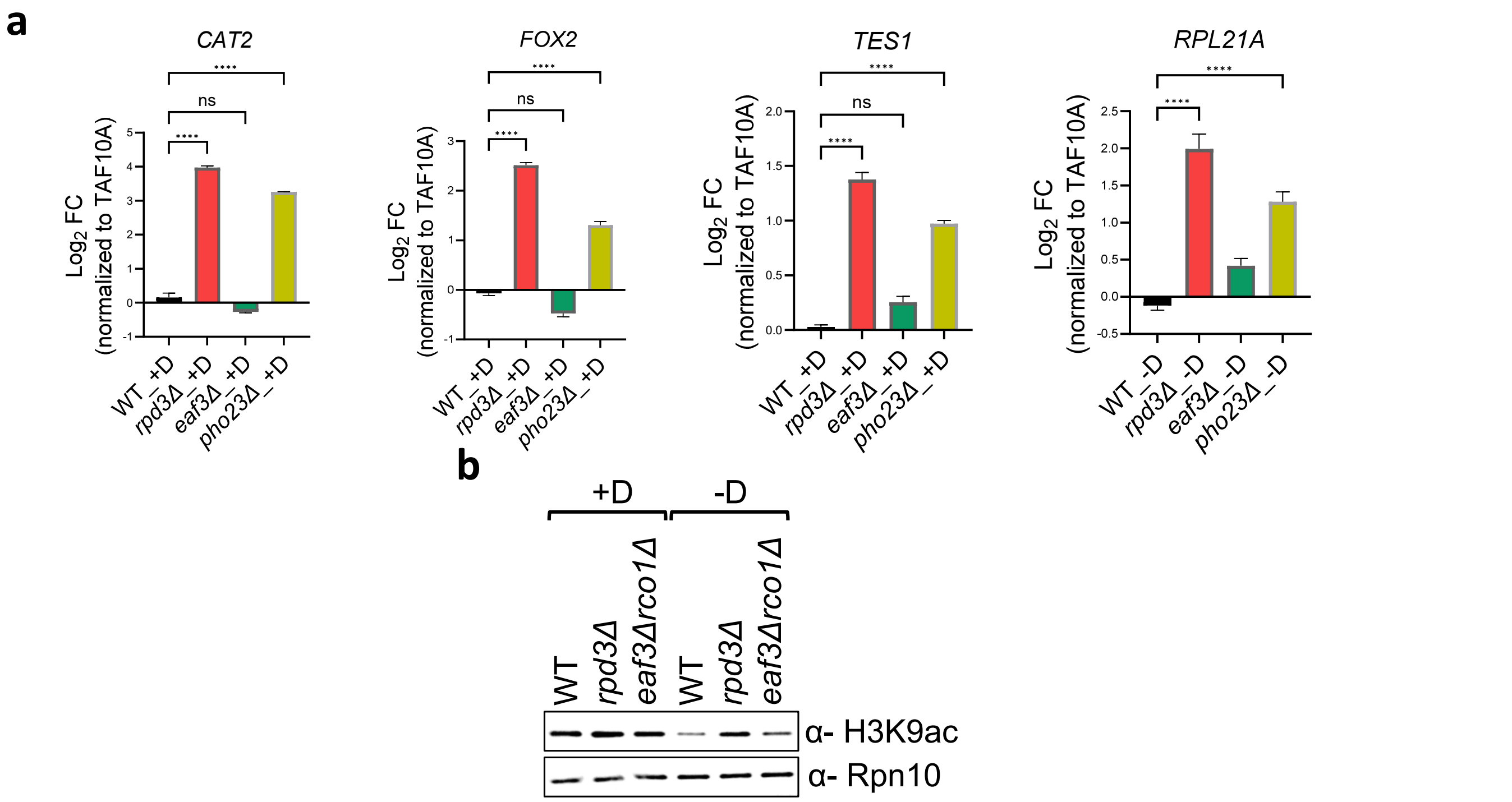

Supplementary figure S5: (a) qPCR analysis of representative genes in WT, *rpd3Δ*, *eaf3Δ*, and *pho23Δ*. Gene expression levels were normalized to an internal control, *TAF10A*. Ordinary one-way ANOVA was used to calculate statistical significance. n=3, ns>0.9999, ***p=0.0001, ****p<0.0001. The error bars depict the SEM. (b) Western blot showing H3K9ac levels in WT, *rpd3Δ*, and *eaf3Δrco1Δ* strain in +D and -D. Rpn10 was used as a loading control.

Primer	Sequence 5' to 3'
CAT2_RT_F	GGCGGAAAAGCCGAACAAAT
CAT2_RT_R	TTCGGGCACGGGTAATGATG
POX1_RT_F	TGAGACAGACTTGCGGAGGA
POX1_RT_R	CTGAACCACCCAGTCGTCAT
RPL21A_RT_F	TTTCCAAACATTCGGTTATG
RPL21A_RT_R	ATCTGTTACCGACCATCTTG
TAF10_RT_F	ATATTCCAGGATCAGGTCTTCCGTAGC
TAF10_RT_R	GTAGTCTTCTCATTCTGTTGATGTTGTTGTTG
CRC1_RT_F	AGATGTCAAAATGGACAAGC
CRC1_RT_R	CCCTTAGAAGATGTTTGCAG
FOX2_RT_F	AGCATCCATATCAACTCTCG
FOX2_RT_R	GCGTTATTGACCAAGATGTC
RPS18A_RT_F	ACGCTTTGACCACTATCAAG
RPS18A_RT_R	TCTTCTACCAGTGGTCTTGG
TES1_RT_F	AACCTCGATGAGCAAGTATG
TES1_RT_R	GGTGAAAGTAAATCGTGTGG

Table S2: qPCR primers
